# Walking and scuba diving assisted amphibious exoskeleton robots: the designing of power assist control and myoelectricity based wearers' fatigue evaluation

**DOI:** 10.3389/fnins.2024.1472184

**Published:** 2024-09-27

**Authors:** Shuai Wang, Yinuo Yao, Xuwei Lu, Pengjie Qin, Xiangyang Wang, Jianquan Sun, Chunjie Chen, Xinyu Wu

**Affiliations:** ^1^Navy Submarine Academy Rescue and Salvage Department, Qingdao, China; ^2^School of Information Engineering, China University of Geosciences Beijing, Beijing, China; ^3^Center for Intelligent Bionic, Shenzhen Institute of Advanced Technology, Chinese Academy of Sciences, Shenzhen, China; ^4^Department of Computer Science, University of Sydney, Sydney, NSW, Australia

**Keywords:** exoskeleton, EMG, neural network, wearable device, adaptive control, transferable scenario

## Abstract

Exoskeleton robots have the potential to augment human motor capabilities. however, current control strategies often require task-specific control laws tailored for different scenarios, which limits the applicability of exoskeletons. In this study, we propose a control strategy for exoskeleton robots that is adaptable across various scenarios. We employ adaptive oscillators (AO) with feedback control to rapidly estimate the wearer's motion phase and subsequently provide torque assistance to the wearer's hip joint based on a TCN-LSTM model. During experiments, we collected surface electromyographic (sEMG) signals from the tibialis anterior, gastrocnemius, and rectus muscles of seven groups of subjects performing treadmill walking and inclined treadmill exercises. We utilized the short-time Fourier transform to extract frequency characteristics of the signals and statistically analyzed the rate of frequency change in each muscle group under different strategies. The results indicate that when wearing the exoskeleton, the overall muscle frequency changes more slowly, suggesting that subjects can maintain activity for a longer duration before fatigue sets in. This control strategy effectively reduces the energetic cost of lower limb work for the wearer and enhances the exoskeleton's versatility in various applications.

## 1 Introduction

Exoskeleton robots have now been widely applied in our daily lives, making carrying loads more effortless, enabling athletes to run faster, and allowing patients with hemiplegia to move again (Zhang and Huang, 2018; Li et al., 2019; Choi et al., 2018; Wu et al., 2021). Exoskeleton robots have demonstrated their capability to provide assistance to individuals during specific tasks. However, the emerging problem, is that different tasks and scenarios often require control objectives that match their needs (Yan et al., 2015). For instance, in the case of lower limb assistive exoskeletons, the significant cost associated with exoskeleton robots highlights the importance of identifying a control strategy that exhibits strong robustness and can be seamlessly integrated into various task scenarios. This holds considerable engineering significance. What, then, limits the transferable of exoskeletons? The critical challenge lies in the design of the exoskeleton controller. The control framework of exoskeleton robots is divided into high-level, middle-level, and low-level (Tucker, 2015; Narayan et al., 2023). The high-level layer estimates the wearer's motion intentions and decision-making; the middle level specifies sub-task control laws, and the low-level layer completes control through specific actuation mechanisms. The complete control process is a tightly integrated procedure; the challenge lies in the fact that the main differences for various tasks are centered on distinct motion rhythms and specific assistive torques. If we can bypass the process of motion intention decoding in the advanced controller and find a universal control strategy, it could significantly enhance the applicability of the exoskeleton. In this paper, we extract phase information from motion signals, collect joint torque data sets of walking actions, and use transfer learning methods to deploy to target tasks with a small amount of target data sets. During continuous task transitions, motion rhythm is the foundation of assistive mode switching; motion rhythm unifies the wearer's gait, and gait is a description of continuous motion events within a motion cycle. For wearable devices, such as exoskeleton robots, accurate gait events are particularly crucial for providing precise assistive force (Young et al., 2017; Kang et al., 2020; Murray et al., 2015). In Kawamoto et al. (2015), it is pointed out that asymmetric gait can lead to the following negative effects: (1) a decrease in dynamic control balance capabilities, (2) increased energy consumption, (3) a reduction in overall activity levels. In the rehabilitation exoskeleton assistive system of Aguirre-Ollinger et al. (2019), the authors use the kinematic trajectory of a healthy wearer as a reference trajectory. However, the gait characteristics of patients may differ significantly from those of healthy wearers, and compensatory movements of the hip joint by healthy wearers result in a trajectory that may not be the optimal gait trajectory relative to the patient (Qian et al., 2022). Similar time-based estimation methods match different sensor outputs with gait events (Nazmi et al., 2019; Hao et al., 2019), which although simple and practical, cannot be used in variable speed and model scenarios and have limitations. Adaptive oscillators (AO) are a model-free phase estimation method (Seo et al., 2015), which can use past motion trajectories to predict the next action, thereby estimating the gait cycle (Murray et al., 2015). This method is used to describe gait events with continuous cycles and has been proven to possess strong adaptability and robustness.

In the realm of exoskeleton robotics, traditional models have predominantly focused on facilitating a singular type of motion (Liang et al., 2024; Huang et al., 2019; Zhang et al., 2024). An active assistance control approach, termed assist-as-needed (AAN), has been posited (Shahbazi et al., 2018). However, the development of a universal AAN algorithm has a formidable challenge. Torque assistance control can generally be categorized into model-based and feedback-based control strategies. For instance, Zhang et al. (2017) and Franks et al. (2021) have employed a Human-in-the-loop controller, which, while effective, necessitates an extended learning period and lacks the adaptability to real-time motion variations. Another approach, based on model predictive control (MPC), has achieved on-the-fly transitions between modes of assistance by leveraging physically-driven models (Aguirre-Ollinger et al., 2019), but encounter nonlinear scenarios, it can be difficult to build an accurate model (Liang et al., 2022). Data-driven methods, as seen in Caulcrick et al. (2021), identify the mapping relationship between phase and assistive force, offering a viable solution that eschews complex sensor suites (Molinaro et al., 2024). However, these approaches typically require extensive datasets for training, which may incur significant costs in specific tasks. Research has demonstrated that walking and waterstroke motions share substantial similarities (Wang et al., 2020), both being describable as repetitive cyclical movements achieve the potential of transferable such analogous motion patterns is promising. In this work, to achieve controller nesting, we have designed a deep network model based on transfer learning strategies, employing a TCN-LSTM-based recurrent neural network architecture, which significantly enhances the representational capacity for sequential data (Hochreiter and Schmidhuber, 1997). Leveraging above-mentioned network models, our approach enables adaptive control for a variety of periodic motions, significantly reducing the reliance on large datasets and based on this, we believe that through this transfer learning framework, knowledge acquired in one domain (such as walking) can be transferred to another domain (such as deep space exploration), enabling rapid adaptation and enhancement of performance.

Based on the above problems, in this work, we designed an assistive evaluation framework that can be applied to amphibious exoskeleton.

(1) An adaptive Ao controller is proposed, which can effectively converge and quickly track changing periodic signals to achieve fast scene switching; In addition, after the scene switch is identified, a multi-step prediction strategy based on the Ao model is designed, which effectively accelerates the convergence speed through the recurrence of the current cycle to achieve fast help.

(2) We propose a deep network model based on TCN-LSTM neural network architecture, which enhances the representation capability of sequence data. By utilizing transfer learning strategies, we explore the potential for nesting circular-like movement patterns, such as walking and swimming, because of their inherently cyclical nature. Our model adapts to similar periodic motion with minimal data set requirements, so as to realize adaptive auxiliary control of lower limb periodic motion for multiple scenes with limited data.

(3) We have designed an sEMG signal-based assistive assessment experiment. By statistically analyzing the frequency change rate of the sEMG signals before and after each batch of experiments, it is possible to determine the degree of fatigue of the muscle group in question. Through control experiments, we can demonstrate whether our exoskeleton's assistance strategy can make the subjects feel more relaxed.

The structure of this paper is as follows: the second section describes the data set and experimental platform design in detail, the third section introduces the adaptive oscillator and the hip moment estimation network, the fourth section shows the experimental results, and the fifth section summarizes the work.

## 2 Design

This section will be composed of several parts from the experimental use of lower limb exoskeleton robot design, data acquisition, control framework: adaptive oscillator (AO) and long short-term memory network and time Convolutional network (TCN-LSTM) control framework, aiming to introduce the hardware to software content of this work.

### 2.1 Experimental platform

To Meet the needs and challenges of underwater and land multi-scenario applications, the following principles need to be determined (Wang et al., 2024, 2023): First, the weight of the exoskeleton should be designed to be light, reduce the environmental impact caused by counterweight problems, and improve underwater operation mobility; Secondly, the exoskeleton should have ergonomic characteristics adapted to different lower limb lengths; Finally, the exoskeleton should be combined with the motion mode to limit the actual joint drive, such as the hip Angle of the water stroke action should be between [-40°, 40°], which is based on the human kinematics (Nakashima et al., 2015) to ensure safety.

**Figure 1:**
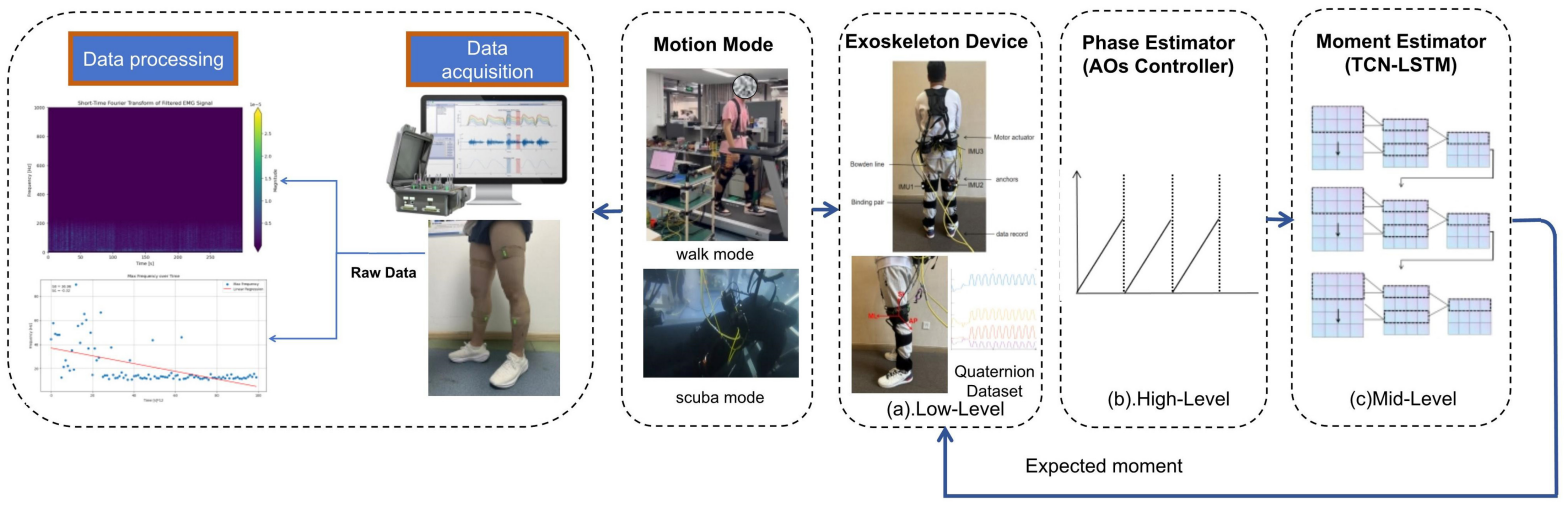
Exoskeleton assistance frame. From left to right is sEmg acquisition and analysis, the application scenario selection, exoskeleton equipment and sensor position; Through the motion sensor placed in the hip and knee, the attitude quaternions are output to the AOs controller. The Aos controller will first change the data in the frequency domain and high-pass filtering, and then realize the phase division. Through pre-training, the TCN-LSTM network outputs a desired torque to the exoskeleton using the input phase, and the wearer can actively adjust the power sensation according to their own stiffness to achieve a closed loop of power control.

In this paper, we designed a wire-driven flexible lower limb exoskeleton (Xiangyang et al., 2024) as Figure 1. The executive motor is placed on the back of the exoskeleton, and the joint is lifted by an inner Bowden cable to achieve power. The motor and driver used in this system are self-developed products, and the motor Angle can be output through the embedded encoder. The motion sensor is based on Wit-9073 (Witmotion Company), and the attitude quaternion is output. We chose to place sensors on the back, left knee joint, and right knee joint of the robot as feature sources, which have been proven to be the most important locations for determining classification accuracy (Sang et al., 2018). The inertial sensor architecture consists of a Speedgoat real-time target machine, a high-precision nineaxis sensor, and a power circuit. The inertial sensor establishes real-time communication with the controller through the EtherCAT bus.

The sampling frequency of the sensor is configured to be 1,000 Hz, and it will be turned on simultaneously after initialization to ensure information synchronization. The position of the sensor is fixed. To ensure the accuracy of the experimental results, we conducted several experiments and readjusted the position of the sensor before each experiment to prevent the sliding or instability of the sensor from affecting the experimental results.speedgoat is a system developed based on matlab-simulink, which provides a variety of communication protocol interfaces. The motor and sensor use ethercat bus for communication. Through the DataInspector interface inside simulink, signals of each channel can be read online to complete data acquisition.

The electromyography (EMG) sensors utilized are products from Delsys Corporation. Each sensor has an extremely light weight of only 14 grams, and they can be worn without the need for electrode pads. They are connected to an upper computer that has built-in filtering and noise reduction functions. Moreover, they have interfaces for kinematics and motion capture, and the collected data is saved in XML format for later processing.

### 2.2 Data collection

In this experiment, in order to achieve the control goal under multiple scenarios, behavioral data under land and underwater scenarios were collected. The training data of land walking were collected from 8 subjects (age: 27 ± 5 years old, weight: 77 ± 32 kg, height: of 1.69 ± 0.11 m) in the paper (Luo et al., 2024). All participants had a walking gait, and there was no lower limb injury in the past 6 months. The experimenters walked at a speed of 0.75 m/s on the treadmill, and the walking method was in accordance with the habits of the subjects. The inertial sensor recorded the data at a frequency of 200 Hz.

Data of underwater freestyle swimming come from Swimsuit fluid simulation platform developed by Tokyo Institute of Technology, Japan (Liew et al., 2023). Human swimming is an unsteady flow process, involving fluid dynamics (CFD) analysis. The former obtains experimental data through image velocity measurement (PIV) (Equation 1). Where Δ*x* represents the shift of the marker in Δ*t* time, PIV acquisition device generally includes multiple high-speed cameras, a laser (Nd:YAG lasers are commonly used) controlled by an optical system, and a precision synchronizer, which coordinates the operation of the cameras and the laser, ensuring precise timing and synchronization for the processes involved.

CFD is a numerical method to solve the Navier-Stokes equation describing fluid dynamics (Zawawi et al., 2018). The computational domain is divided into a large number of grids, and the velocity and pressure of the fluid are calculated based on the grid, so as to realize the discrete space. The mesh size needs to be less than the minimum vortex length, i.e., the Kolmogorov length scale η, which defind as (2), where *v* is the kinematic viscosity of the fluid, *U* is the characteristic velocity of the fluid, and *L*is the characteristic length of the fluid.


(1) $$\begin{array}{l} u\left(x,t\right)=\frac{\Delta X\left(x,t\right)}{\Delta t} \end{array}$$

(2) $$\begin{array}{l} \eta ={\left(\frac{v^{3}L}{U^{3}}\right)}^{\frac{1}{4}} \end{array}$$

**Figure 2:**
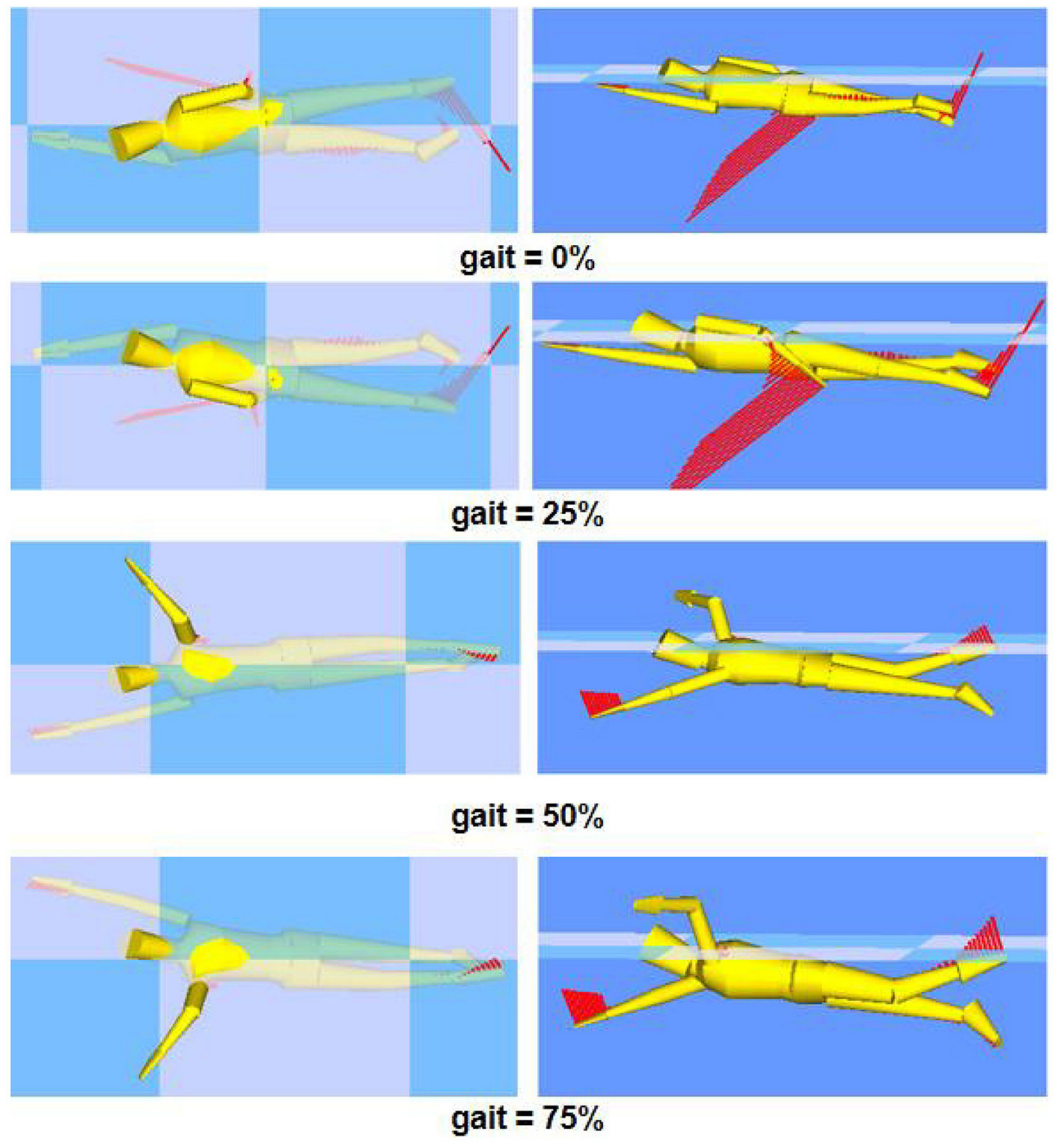
Different phases of water striking action schematic diagram.

The Swumsuit takes into account common fluid properties such as added mass and unsteady fluid forces. Swimsuit has interfaces for human joint position, characteristics and kinematic Settings, and the simulation performance error of predicting the time change of fluid force under the determined fluid force coefficient is less than 7.5%, which is generally satisfactory. We collected the body information as follows: (height: 1.65 m, weight: 50 kg), (height: 1.68 m, weight: 53 kg), (height: 1.75 m, weight: 64.5 kg), (height: 1.80 m, weight: 75 kg), with human data of different lower limb proportions, their movement trajectory was set as a standard freestyle movement, and the feet alternated up and down twice for one movement cycle, and the kinetic data was collected at a frequency of 100 Hz. Figure 2 shows the out-of-phase motion posture in one cycle.

## 3 Method

### 3.1 AOs-based high-level controller

Adaptive oscillator was proposed by Righetti et al. (2006). Its purpose is to continuously estimate the periodic input signal characteristics (frequency, phase, amplitude). By learning the target signal, the system can still maintain a synchronous oscillation frequency with the previous one after removing the input after learning (Righetti et al., 2006).

**Figure 3:**
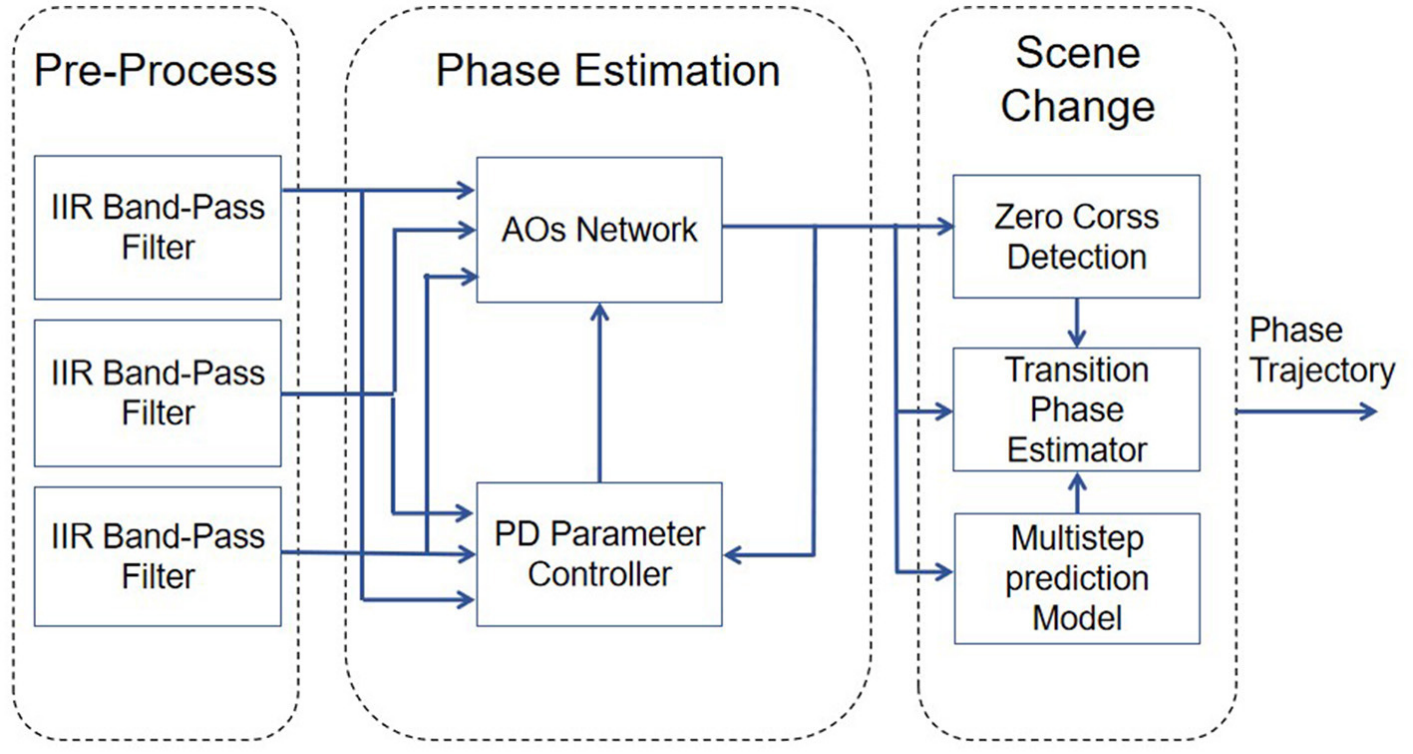
Structrue of AOs controller.

As shown in Figure 1, AOs will estimate the gait phase in real time from the input kinematic signal. The controller consists of three modules (Figure 3). The input preprocessing module, the core of which is IIR filter; Phase Estimation module is composed of AOs Network and adaptive PD adjustment module for AOs parameters. The Scene Change module applies zero-crossing detection and multi-step prediction model output estimation phase to the output of the previous step.

#### 3.1.1 Pre-process

An infinite impulse response (IIR) bandpass filtering mechanism is used to suppress the high frequency noise and DC bias in the signal. The filter is designed with a specific passband frequency range, i.e. 0.2–20 Hz, to preserve the main low-frequency dynamic features during motion. Subsequently, in order to mitigate computational discrepancies stemming from variable data magnitudes, as well as to avert convergence rate impediments in modeling due to possible numerical overflows within the signal, the signal amplitude is standardized and adjusted to the range of [-1, 1] to facilitate the standardized input of subsequent processing steps.

A consistent preprocessing strategy is also adopted for the Angle of the encoder output and the quaternion features obtained from the motion sensor. However, according to the characteristics of angular data and kinematic data, the parameters of amplitude normalization are adjusted respectively, so that the maximum response value of each signal can be close to the unit amplitude. Through this method, the aim is to achieve effective normalization of all kinds of signals.

#### 3.1.2 Phase estimation

In Section 3.1, we introduce the application background of AO, and the mathematical derivation of AO will be introduced in detail in this section. The core of AO is to adjust the phase of the oscillator according to the error between the estimated frequency and the true frequency. When both frequencies are closed, the oscillator's signal is synchronized with the input signal. Firstly, the kinematic signal u(t) is estimated as sine function superposition based on Fourier transform:


(3) $$\begin{array}{l} û\left(t\right)=\alpha_{0}\left(t\right)+\overset{N}{\underset{i=1}{\sum }}\alpha_{i}\left(t\right)\sin\left(\phi_{i}\left(t\right)\right) \end{array}$$

In the given equation, û(*t*) represents the reconstructed signal of the oscillator, α_*i*_(*t*) and ϕ_*i*_(*t*) denote the amplitude and phase of the i-th harmonic, respectively, while α_0_(*t*) is an integrator used to learn the offset of the oscillator signal. Additionally, the characteristics of the signal can be specifically calculated through the following formula:


(4) $$\begin{array}{l} {\dot{\phi }}_{i}\left(t\right)=i\omega \left(t\right)+\frac{k_{\phi }e\left(t\right)}{\sum_{j=0}^{N}\alpha_{j}\left(t\right)}\cos\left(\phi_{i}\left(t\right)\right) \end{array}$$

(5) $$\begin{array}{l} ẇ\left(t\right)=\frac{k_{\omega }e\left(t\right)}{\sum_{j=0}^{N}\alpha_{j}\left(t\right)}\cos\left(\phi_{1}\left(t\right)\right) \end{array}$$

(6) $$\begin{array}{l} {\dot{\alpha }}_{i}=k_{\alpha }e\left(t\right)\sin\left(\phi_{i}\left(t\right)\right) \end{array}$$

(7) $$\begin{array}{l} {\dot{\alpha }}_{0}=k_{0}e\left(t\right) \end{array}$$

(8) $$\begin{array}{l} e\left(t\right)=u\left(t\right)-û\left(t\right) \end{array}$$

Where i is the harmonic number, ω(*t*) is the base frequency estimated by the oscillator, and *e*(*t*) is the error between the oscillator's estimated output and the actual output, which represents the degree of convergence of each variable to the input. Kω, *K*_0_, Kα are learning rate coefficients, which determine the learning speed of the model. The PD Parameter Colltroller adjusts the above parameters. With the PD controller, the AOs model can adapt effectively without flying when facing environment-task (frequency) changes. This ensures security and completes adaptive task switching, Enhancing model transferability.

According to the experiment, we find that the kinematics curve of human walking task is similar to sine wave, and setting n to 2 will have better tracking effect. In underwater fetching tasks, because of the strong nonlinearity, setting n to 3 will have a better effect.

### 3.2 LSTM-TCN-based mid-level controller

#### 3.2.1 Overview

In the first section, we explained the feasibility of data-driven exoskeleton power, and this section will specifically introduce the Mid-Level architecture based on the LSTM-TCN model.

Torque regression is a common sequential task, and a large number of studies have proved that recurrent neural networks (RNN) have a good performance in dealing with such problems (Molinaro et al., 2022; Mundt et al., 2020; Dorschky et al., 2020). LSTM structures control the flow of information and learn long-term dependence of long and short term memory units by designing input gates, forgetting gates and output gates. Temporal and spatial convolutional networks (TCN) employ causal convolution and extended convolution to capture local features and process translation invariance in data. Compared to the traditional CNN framework, TCN can also capture the causal relationship of the sequence, which has significant advantages when dealing with time series data (Pratap et al., 2024), which making TCN perform well in short-term features (Molinaro et al., 2024).

In addition, TCN has simple structure and advantages in efficiency, and adding residual connections can effectively avoid the problem of gradient disappearance (Gan et al., 2021; Bai et al., 2018). However, in the capture of long-term relationships, because TCN can only improve the learning ability by increasing the depth of the network, no state information of previous layers is retained between each convolutional layer. Compared with the unique gating mechanism of LSTM, the latter is more suitable for processing data with high contextual correlation.

**Figure 4:**
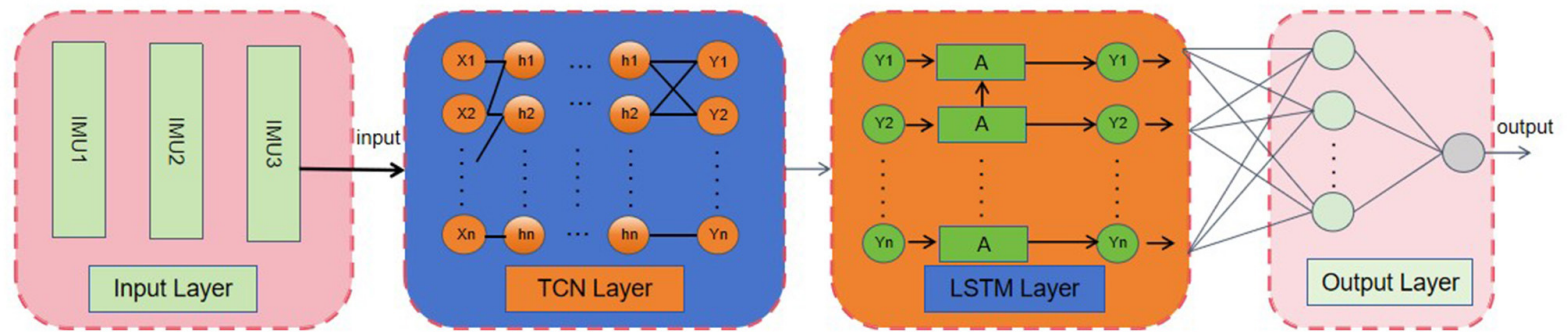
TCN-LSTM network overall flow chart of model.

In this paper, we construct a TCN-LSTM model to predict joint torques based on phase and historical data. The model consists of three parts: input layer, feature extraction layer and output layer. The input layer manages the data input of each channel; The feature extraction layer is composed of three layers of TCN, which have the same number of filters and kernel size, and the expansion factor is 1,2,4. dropout is added after each convolutional layer to avoid overfitting. After the TCN layer, time features are extracted through two layers of LSTM model. Through the above series of nonlinear transformations of the input layer data, the deep features are extracted. The output layer is composed of two fully connected layers, and the result is predicted by dimensional transformation. This model not only contains the understanding of medium and long-term dependencies and complex patterns of time series data, but also has a stronger ability to express short-term features. The working flow chart of this model is shown in Figure 4.

#### 3.2.2 Temporal convolutional network (TCN)

**Figure 5:**
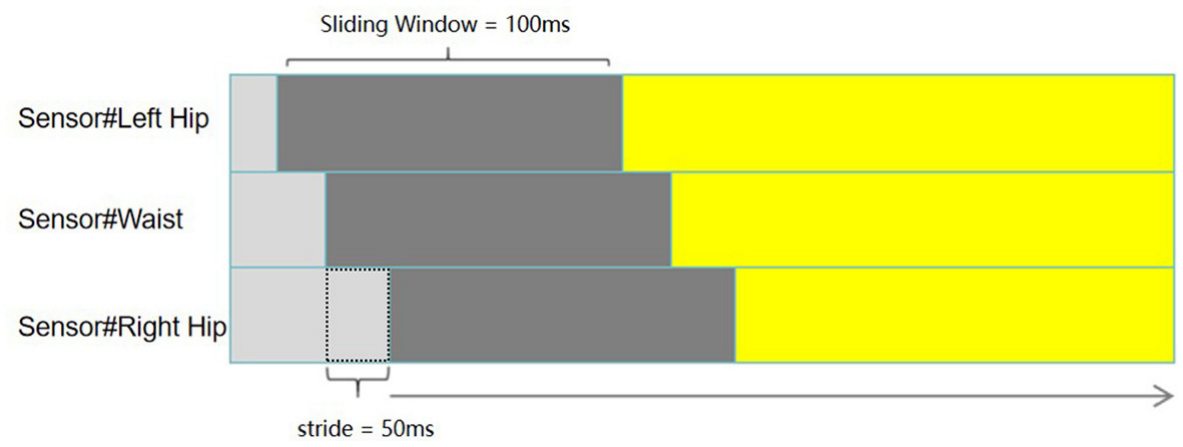
Sliding windows process. The window size is 100 ms and the window stride is 50 ms.

The application of time series data in neural networks requires data to be transformed into sequences. Generally, the sliding window method is adopted to segment the data set and obtain an input sequence as the input feature. Input features and output labels of the time series regression problem need to be presented and defined (Benson et al., 2020) for the TCN-LSTM model. The time window is equal to the number of samples in one period to predict the next observation results, and the input and output of the data set are divided through a sliding window. Figure 5 shows the sliding window process for our one-step time series.

Sequence modeling tasks require the ability to capture continuous data, understand the coherence of the data along its expansion direction, and comply with causal constraints, that is, the prediction of the current moment cannot be based on the input of the future moment (Wei et al., 2024). TCN is an advanced sequence modeling framework designed for processing sequence data with causal relationships. Compared to traditional recurrent neural network (RNN), TCN provides a novel way to capture dynamic features in time series while following the principle of causality.

**Figure 6:**
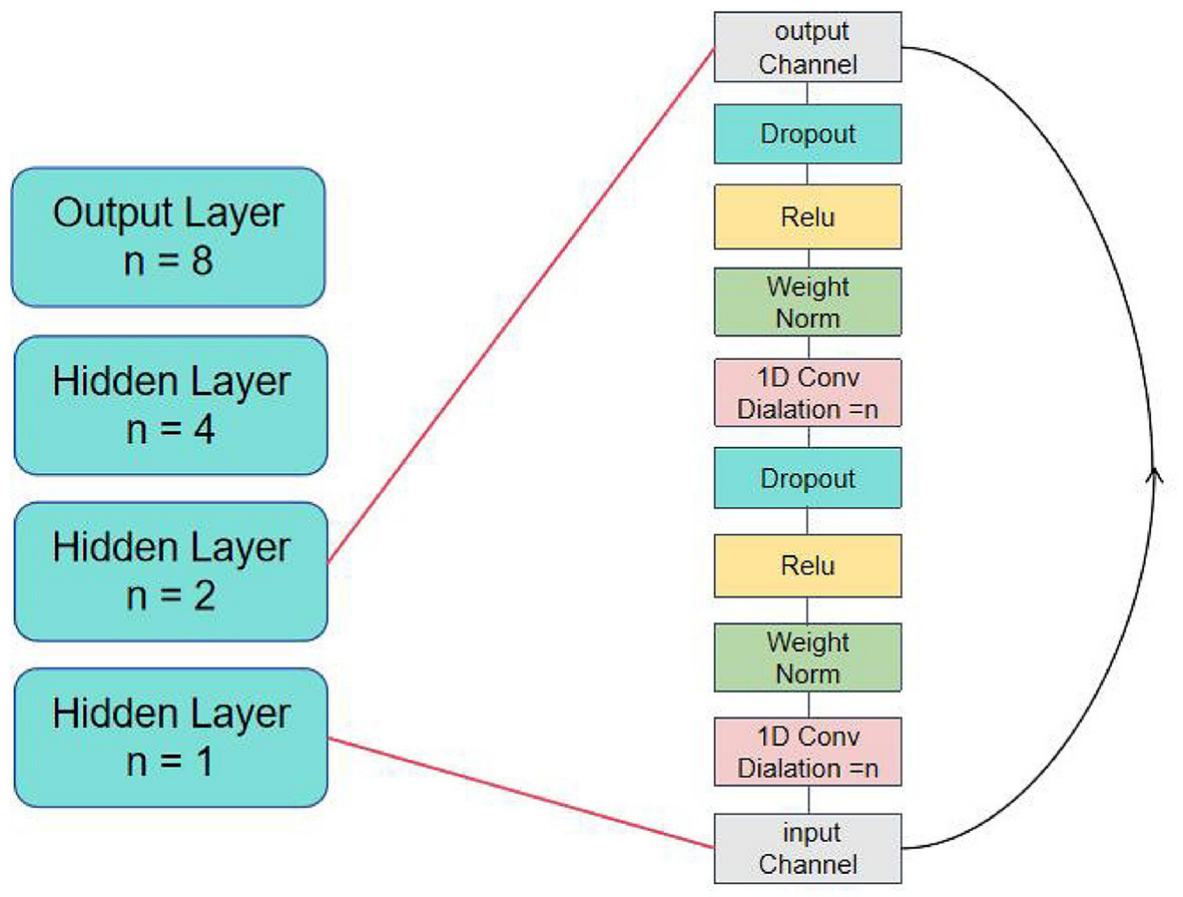
The struct of TCN network.

TCN achieves these requirements through a range of innovative technologies. Firstly, TCNs uses causal convolution, a special convolution operation that ensures that the direction of the sequence follows the predicted causal law. Secondly, TCN uses extended convolution to increase the model's receptive field, which enables the network to capture longer time dependencies with fewer parameters (Cheng et al., 2021). For a one-dimensional input sequence *X*, the extended convolution operation *F* can be expressed by the Equation 9. Finally, TCN uses residual connection to alleviate the problem of gradient disappearance in deep network training and improve the learning ability of the model (Zhou et al., 2019). Specifically, the TCN structure is shown in Figure 6, which contains three hidden layers, each of which is composed of two expanded convolution layers, two batch normalization layers, two dropout, two Relu activation layers and a residual link layer.


(9) $$\begin{array}{l} F\left(s\right)=\left(x{*}_{d}f\right)\left(s\right)=\overset{k-1}{\underset{i=1}{\sum }}f\left(i\right)\cdot x_{s-d\cdot i} \end{array}$$

#### 3.2.3 Long short-term memory network (LSTM)

LSTM network is A variant of RNN structure, which adjusts hidden neurons in RNN into a special gate structure, enabling it to capture long-term dependencies in time series data (Molinaro et al., 2022). This gate structure corresponds to the “A” module in Figure 4. The internal structure of concrete containing unit state *C*_*t*_, input door *i*_*t*_, output door *o*_*t*_ and forget *f*_*t*_. The flow of LSTM information depends on the special internal state (Shahbazi et al., 2018). The current input *X*_*t*_ will concat operation with the output *h*_*t*−1_ of the previous layer. *f*_*t*_ selectively forgets the information of the previous moment to control the loss degree of *C*_*t*_. *i*_*t*_ selectively records the information of *X*_*t*_ into *C*_*t*_; *o*_*t*_ controls the amount of information corresponding to external output *h*_*t*_ at the current time. Let *X*_*t*_ be The input at the current time, and the output through the network is *h*_*t*_. The formula for calculating LSTM is as follows (10) to (15) (Zhou et al., 2019).


(10) $$\begin{array}{l} f_{t}=\sigma \left(W_{f}\cdot \left[h_{t-1},x_{t}\right]+b_{f}\right) \end{array}$$

(11) $$\begin{array}{l} i_{t}=\sigma \left(W_{i}\cdot \left[h_{t-1},x_{t}\right]+b_{i}\right) \end{array}$$

(12) $$\begin{array}{l} {\tilde{C}}_{t}=\tanh\left(W_{C}\cdot \left[h_{t-1},x_{t}\right]+b_{C}\right) \end{array}$$

(13) $$\begin{array}{l} C_{t}=f_{t}⊙C_{t-1}+i_{t}⊙{\tilde{C}}_{t} \end{array}$$

(14) $$\begin{array}{l} o_{t}=\sigma \left(W_{o}\cdot \left[h_{t-1},x_{t}\right]+b_{o}\right) \end{array}$$

(15) $$\begin{array}{l} h_{t}=o_{t}⊙\tanh\left(C_{t}\right) \end{array}$$

## 4 Result

### 4.1 Model evaluation

**Figure 7:**
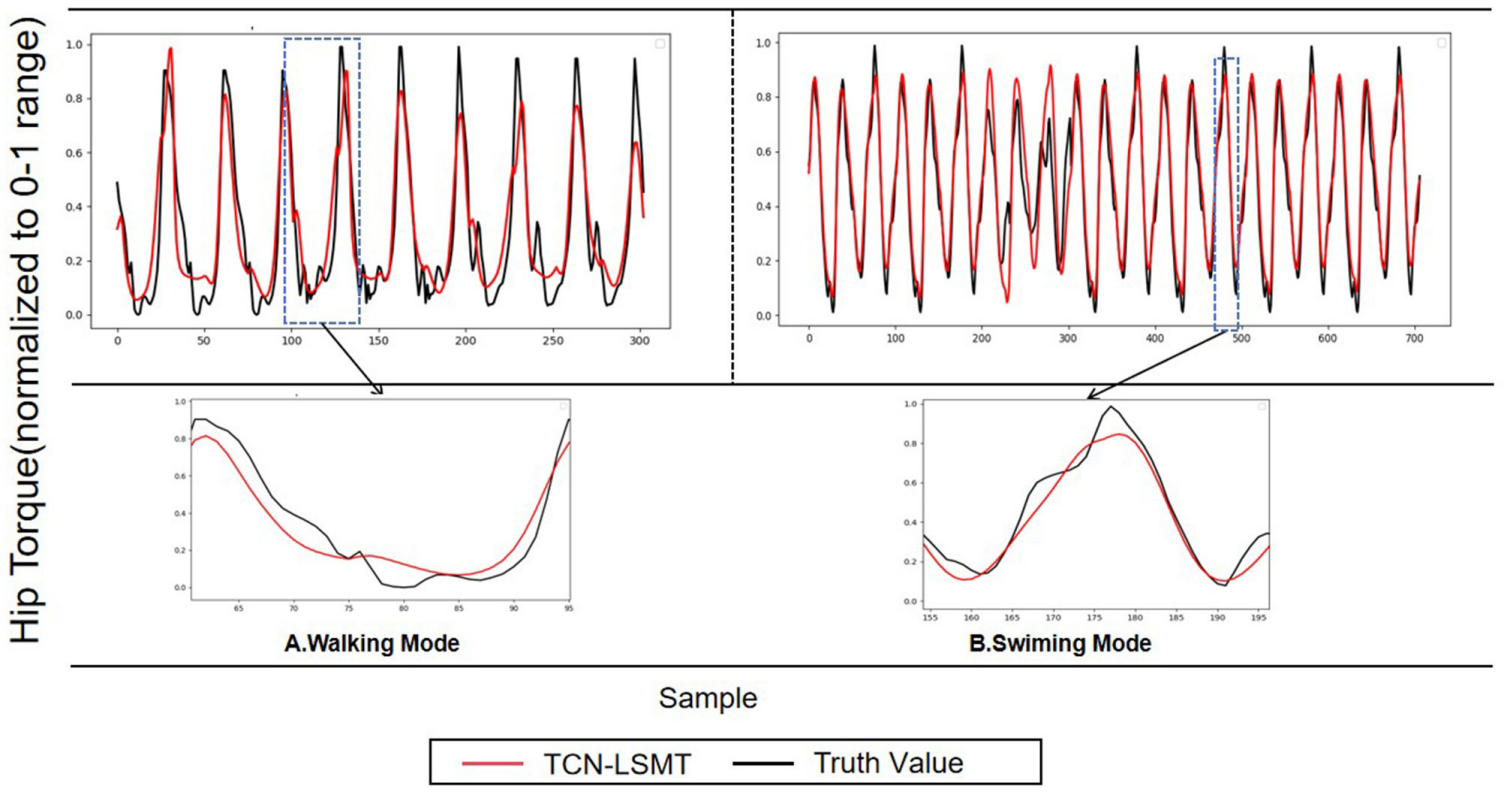
Estimating representative hip moments have the TCN-LSTM model, and forecast the output of the hip joint torque (red line), truth value (black line).

In Section 2.2, we introduced the collection process of the data set. In off-line training and testing, we divided the data according to 80% training set and 20% test set. TCN-LSTM network is composed of one LSTM layer, three TCN convolutional layers, and a fully connected layer. The activation function used is ReLU, and the network is trained using stochastic gradient descent with a batch size set to 64 and an initial learning rate of 0.001. In off-line testing, we focused on the accuracy of the torque estimate returned by the TCN-LSTM model. Figure 7 shows the predicted stride length of the single sagittal leg of the hip in different modes (Walking, Runing, Swiming) (Red Line), and the true value of each data (Black Line) is also shown.

**Figure 8:**
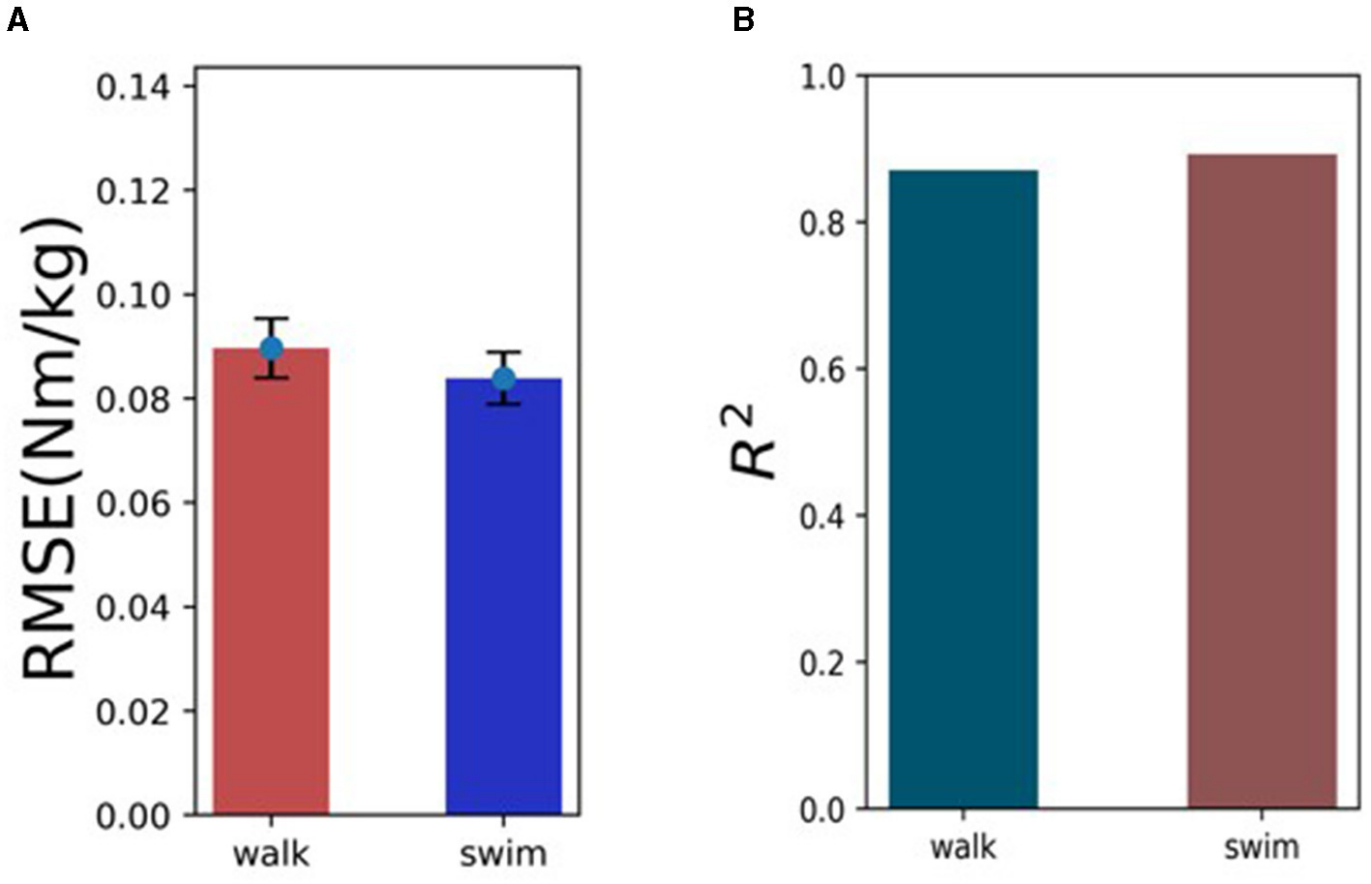
The predictive performance of the TCN-LSTM model across walking and swimming activities, **(A)** highlighting the RMSE and *R*^2^ scores. The RMSE values are 8.9% for walking and 8.3% for swimming (the lower percentages indicating higher accuracy). **(B)** The *R*^2^ values are 0.87 for walking and 0.89 for swimming (the higher values represent better model fit).

The statistical results are shown in Figure 8. The models of walking action (RMSE = 0.089) and swimming action (RMSE = 0.083) show better performance, and the trough in walking mode have certain uncertainties, which may be related to the speed and habits of different subjects during movement. In general, the TCN-LSTM model accurately fits the changes of hip moment in different stages under different periodic movements.

There have been extensive studies on hip torque estimation under offline conditions (Kang et al., 2020; Mundt et al., 2021), but in online applications, dynamic verification under real environment interaction plays a crucial role in evaluating the performance of the control framework. In the following chapters, we will evaluate the helpability of online applications by sEMG.

### 4.2 Evaluation of exoskeleton assisted performance based on EMG signals

Muscle fatigue results in a degradation of physical movement ability, and while the manifestation of muscle fatigue can differ, there are still commonalities that can be identified through statistical features. Historically, researchers have utilized characteristics such as the amplitude of EMG signals and their spectral shift from high to low frequencies. The prevailing perspective is to examine whether there is a significant reduction in the median and mean frequencies of EMG signals over time as key indicative features (Haddad and Mirka, 2013).

The present experimental investigation aims to validate the efficacy and assess the generalizability of a supportive framework. Electromyographic signals were harvested from a cohort comprising seven participants (six male and one female) engaged in locomotion and incline walking on a treadmill at a velocity of 2 m per second. Kinematic trajectories of the hip joint, ascertained during both walking and incline walking, demonstrated analogous attributes; moreover, incline walking was observed to precipitate fatigue in the participants at a more accelerated rate compared to flat walking, potentially augmenting the discernible outcomes within the framework of our validation. The electromyographic data were procured via six channels, each aligned with the rectus femoris, tibialis anterior, and gastrocnemius muscles of the lower limbs. The sensors operated at a sampling frequency of 1000 Hz, and the raw electromyographic signals underwent band-pass filtration within the bandwidth of 10 Hz to 499 Hz to eliminate noise.

**Figure 9:**
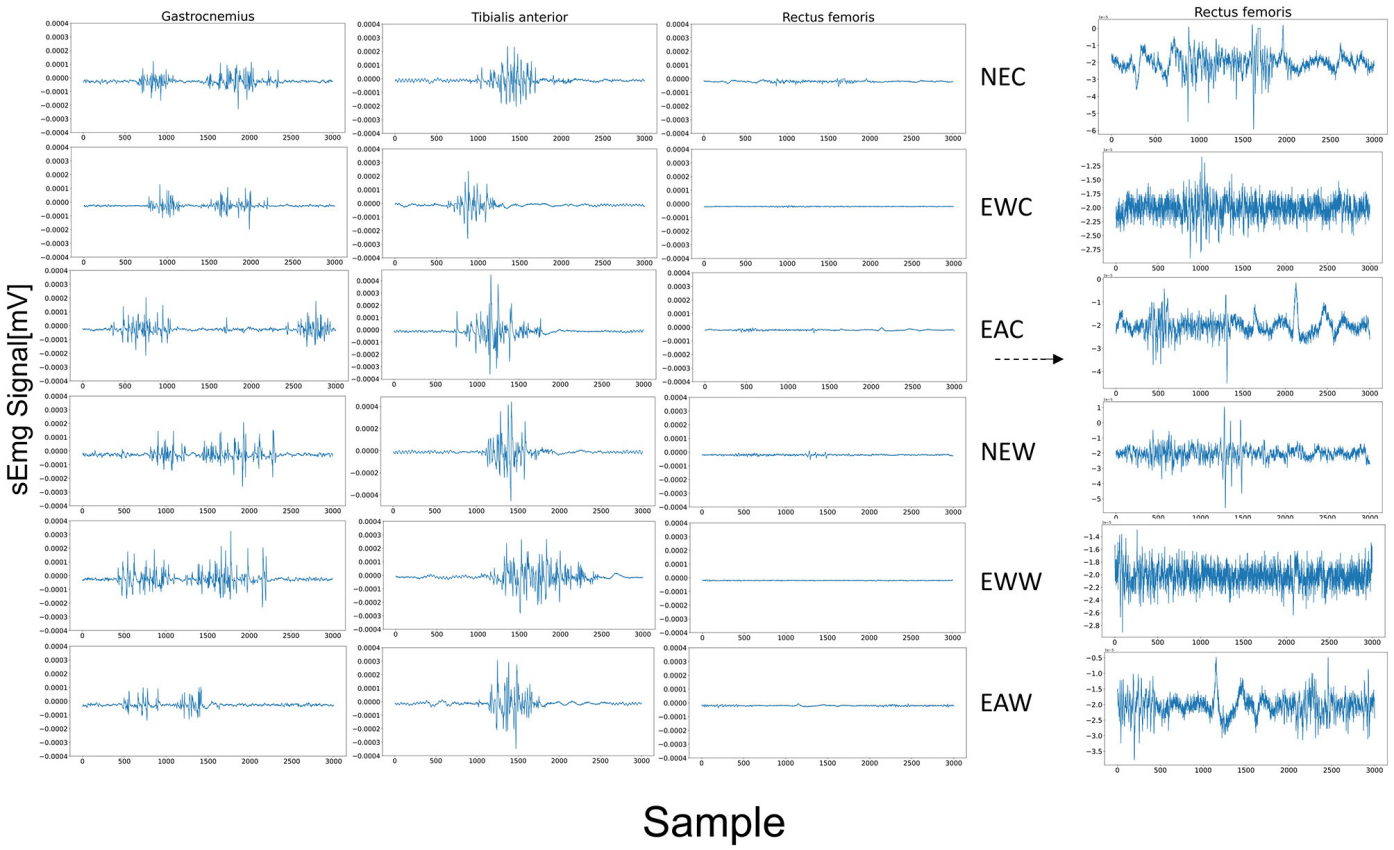
Original sEMG signals of various motion patterns.

In this study, we established a control experiment without the use of an exoskeleton and with the use of an exoskeleton without assistance for the same task. Each experiment lasted for five minutes, with a ten-minute break between groups. As depicted in Figure 9, from top to bottom, they illustrate the raw (EMG) signals for six different motion modes: No Exoskeleton Climb (NEC), Exoskeleton without Assist Climb (EWC), Exoskeleton Assist Climb (EAC), No Exoskeleton Walk (NEW), Exoskeleton without Assist Walk (EWW), and Exoskeleton Assist Walk (EAW). From left to right, the signals represent the Gastrocnemius, Tibialis, and Rectus femoris channels, corresponding to three distinct pathways. Among these, the Gastrocnemius and Tibialis muscles exhibit more pronounced activation, This perhaps indicates that they are more involved in force generation. We also recorded the fatigue node of the subject's subjective feedback, and calculated fatigue quantitatively by using a feature based on median frequency proposed in Chand et al. (2023). The former verified the relationship between muscle fatigue with task load and repetitive movements based on this method, and we combined the them to evaluate the effect of the exoskeleton on the Subject's body.

**Figure 10:**
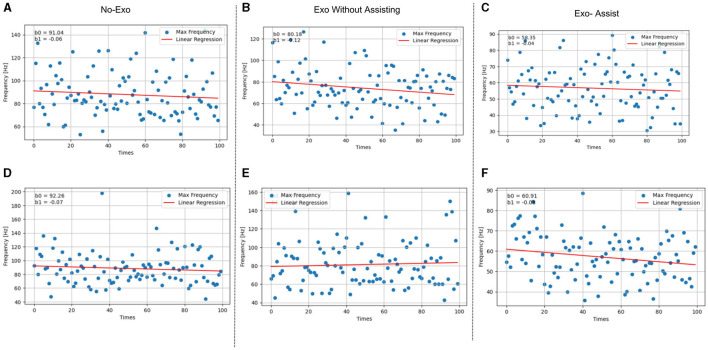
Comparison of sEMG frequency change rates for climbing and walking movements: a visual representation of three modes **(A)** without exoskeleton, **(B)** with exoskeleton without assistance, **(C)** with exoskeleton assistance for climbing, **(D)** without exoskeleton, **(E)** with exoskeleton without assistance, and **(F)** with exoskeleton assistance for walking.

The average value of the input signal was subtracted from each sample point to eliminate the deterministic trend effect. We designed a short-time Fourier transform with a window length of 30,00 sampling points. Accumulated experimental evidence suggests that a sampling size of 3000 points is typically adequate to encompass an entire motion cycle, thereby facilitating a comprehensive analysis of periodic attributes, which is essential for accurate characterization of the motion's periodic nature. and by averaging every three adjacent points, we obtained a frequency curve that varies over time, as shown in Figure 10. Although we collected electromyographic signals from three distinct muscular regions, the gastrocnemius has been established in prior research as the predominant muscle group engaged during lower limb ambulation (De Luca, 1984). Through observation of this dominant muscle group, we can more clearly discern variations in the degree of fatigue.

**Figure 11:**
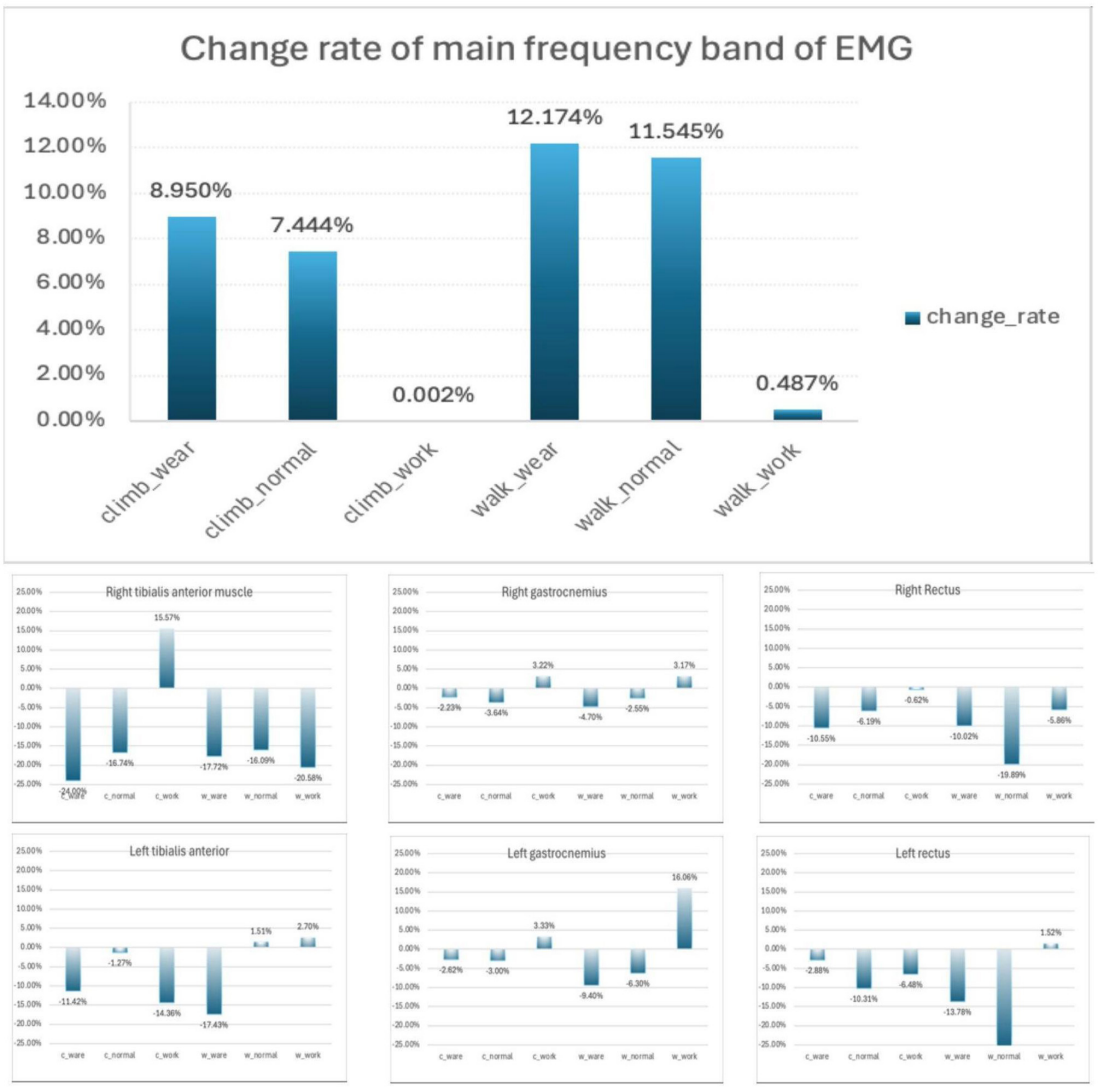
Overall fatigue profile change rate and fatigue profile change rate of each channel.

We conducted a comprehensive statistical analysis focusing on the fatigue contours of electromyographic (EMG) signals for each subject, as depicted in Figure 11. This analysis involved calculating the rate of change in average frequency for each channel under various conditions. Our findings revealed a consistent negative linear relationship between the overall muscle frequency and the progression of fatigue, which aligns with the frequency decay gradient reported by Chand et al. (2023).

Specifically, we observed a significant decline in the rate of muscle frequency change for conditions involving exertion compared to normal conditions: 8.948% for climb_work versus climb_wear, and 7.442% for climb_normal. Similarly, for walking activities, the decline was 11.687% for walk_work versus walk_wear, and 11.058% for walk_normal. These results suggest that the exoskeleton provides substantial assistance, enhancing performance and energy efficiency during tasks that would otherwise be fatiguing when performed without the support of an exoskeleton.

The observed negative linear relationship can be attributed to the manifestation of muscle fatigue, which is reflected in the reduced efficiency of muscle contractions. As muscle contraction efficiency decreases, the muscle's ability to generate force during elongation diminishes, leading to a decrease in the frequency and amplitude of the EMG signals (Haddad and Mirka, 2013). This effect is particularly pronounced in the dominant muscle groups, as they bear a greater load and exhibit more noticeable changes in average frequency.

Conversely, for muscle groups that are less dominant or have a weaker association with the specific movement pattern, the expected negative linear relationship may not be as apparent in our experiments. This variability could be attributed to factors such as the duration of the activity and individual differences in movement habits and biomechanics.

## 5 Disscussion

1. For source datasets with smaller samples, if the target task is complex, it may lead to poor transfer learning effects. In our subsequent research, we plan to delve into and address the challenges of small sample source datasets in transfer learning.

2. While simulated environments provide a controlled and efficient way to collect data, there are still differences between these conditions and those in the real world, For our forthcoming experiments, we intend to incorporate waterproof six-axis force sensors to gather data from authentic environments, thereby leveraging the strengths of both simulated and real-world data to bolster the model's robustness.

3. In the results, we found that the activation of the gastrocnemius muscle during lower limb movement is significantly more pronounced than that of other muscle groups. This could be related to the exercise modality we chose, as well as factors such as the fatigue resistance characteristics of different muscles. In our future work, we will expand our research to cover these areas.

## 6 Conclusion

The control strategy of the exoskeleton robot proposed in this paper can effectively improve the applicability and robustness of the exoskeleton robot in different scenarios by combining adaptive oscillator and deep learning model. The experimental results show that the control strategy not only reduces the lower extremity work cost of the wearer, but also improves the movement efficiency and comfort of the wearer. Specific contributions include: Adaptive AOs controller: Fast and accurate estimation of the wearer's motion phase, providing real-time motion intent feedback for the exoskeleton robot. TCN-LSTM model: Through transfer learning strategy, the model can adapt to different periodic motion patterns, and achieve accurate assistance to hip torque. Multi-scene application verification: Through the analysis of the frequency change rate of EMG signal, the fatigue degree of the subjects was assessed. Through the controlled experiment, the effectiveness of the control strategy in the walking scene in different modes was verified.

Cross-task, cross-scenario operation: The design of the control framework allows the exoskeleton robot to demonstrate powerful performance in multiple tasks and environments, reducing the reliance on extensive data sets and complex sensor suites. Future work will focus on further optimizing control strategies, improving the system's adaptive and user-customized capabilities, and exploring more application scenarios, such as Under analogous kinematic patterns, the deployment of exoskeleton assistance models in environments characterized by unknown gravitational accelerations, such as in deep space, or in conditions with fluid pressure-induced perturbations, such as during deep-sea diving, presents unique challenges. However, employing a transfer learning strategy to formulate a control framework for these contexts is deemed to be promising. In the case of deep space, the unknown gravitational forces may introduce complexities in movement dynamics that are not present on Earth. Similarly, the hydrodynamic pressures in deep-sea conditions can cause disturbances that affect the kinesthetic feedback and load management of exoskeleton systems. Despite these challenges, the transfer learning approach, by its nature, is adept at generalizing across different physical contexts, making it a potent tool for developing robust control frameworks that can adapt to these unknowns.

In addition, the research team will continue to explore the application of deep learning models in the recognition and prediction of different motion patterns to achieve more intelligent and personalized robotic assistance systems for exoskeletons.

## Data Availability

The raw data supporting the conclusions of this article will be made available by the authors, without undue reservation.
